# HSV-1 infections of the prefrontal cortex: regional vulnerability, cellular mechanisms, and neurocognitive consequences

**DOI:** 10.3389/fncel.2026.1887935

**Published:** 2026-09-25

**Authors:** Hannah Chau, Julia Schurr, Jonna M. Leyrer-Jackson, Erin S. Honsa

**Affiliations:** Department of Medical Education, Creighton University School of Medicine – Phoenix Campus, Phoenix, AZ, United States

**Keywords:** CNS, executive function, herpes simplex virus, microglia, neuroinflammation, prefrontal cortex, viral encephalitis

## Abstract

Herpes simplex encephalitis (HSE), caused by herpes simplex virus type 1 (HSV-1), is the most common cause of sporadic viral encephalitis and remains associated with substantial mortality and long-term neurological morbidity despite effective antiviral therapy. Although HSE is classically characterized by involvement of the medial temporal lobes and limbic system, recent clinical, preclinical and neuroimaging studies highlight involvement of prefrontal cortical regions, including the medial prefrontal cortex (mPFC) and orbitofrontal cortex (OFC). The functional consequences of HSV-1 associated injury may be particularly relevant to the persistent executive, cognitive, motivational, emotional, and behavioral abnormalities observed in HSE survivors. However, the mechanisms linking HSV-1 infection to prefrontal injury and subsequent neurobehavioral dysfunction remain incompletely defined. This review examines HSV-1 neuropathogenesis through a prefrontal cortical framework, with a focus on the mPFC and OFC, and evaluates potential mechanisms underlying their vulnerability. Current evidence supports a multistep hypothesis for HSV-1-associated injury involving olfactory and trigeminal neural entry, microtubule-dependent axonal transport, dissemination, and potential regional differences in cellular susceptibility. Further, we highlight cellular mechanisms of HSV-1-associated injury, including microglial and NLRP3 inflammasome activation, astrocytic dysfunction, neurovascular unit disruption, neuronal and synaptic injury, and viral immune-evasion mechanisms that facilitate viral persistence and amplify host-mediated injury. Lastly, we integrate these mechanisms with the functional organization of prefrontal-frontal-limbic networks to consider how regional injury may contribute to persistent cognitive and behavioral dysfunction following HSE. The studies discussed here provide a framework for linking HSV-1-associated prefrontal injury to long-lasting cognitive and behavioral sequelae of HSE, while highlighting critical gaps in our understanding of these HSV-driven mechanisms.

## 1 Introduction

Herpes Simplex Virus (HSV) is an enveloped DNA virus belonging to the *Herpesviridae* family (Ak et al., 2026). HSV is the most common cause of sporadic viral encephalitis from viral members of this family, and without treatment, herpes simplex encephalitis (HSE) has a mortality rate up to 70%, underscoring its clinical significance, timely recognition, and specific antiviral treatment (Lind et al., 2017; Sharma et al., 2025). Unfortunately, even with prompt treatment with acyclovir, mortality from HSE can be up to 30% (Ak et al., 2026). Importantly, survivors frequently experience persistent neurocognitive deficits, including impairments in memory, executive function, and behavior (Sharma et al., 2025).

HSV is initially transmitted through direct contact with infected secretions, including saliva for HSV-1 and sexual contact or perinatal transmission for HSV-2 (Saleh et al., 2026). Following primary infection, the virus replicates in epithelial cells of the mucosa or skin, causing local inflammation, tissue damage, and the characteristic herpes vesicular blisters (Whitley et al., 2007). HSV-1 infection is usually associated with orolabial infection whereas HSV-2 more commonly causes genital and neonatal infections (James et al., 2014). Following primary infection, HSV establishes lifelong latency within sensory ganglia and may subsequently reactivate. Approximately 30% of HSE is attributed to primary infection, while reactivation of latent infection, or reinfection, remains the most common source of HSV entering the brain (Ak et al., 2026). Research shows that HSV can enter the brain by either the trigeminal or olfactory nerve after the primary oropharyngeal infection, or reactivation of viral production and movement along the same neuronal pathway (Niemeyer et al., 2024). In this review, we focus on HSV-1, as it is the most common cause of year-round life-threatening sporadic viral encephalitis worldwide (Ak et al., 2026).

Though all age groups are affected, children and older adults show the highest incidence, as well as the most severe manifestations of HSE, most likely due to initial HSV infection as a child, and waning immunity in older age with reactivation of latent infection (Whitley and Gnann, 2002). HSE typically presents as an acute or subacute encephalitic syndrome, often preceded by nonspecific flu-like symptoms. As the central nervous system (CNS) disease progresses, patients may develop fever, headache, altered mental status, new-onset seizures, focal neurological deficits, and changes in behavior, personality, and cognition (Ak et al., 2026). Fever and confusion are among the most common manifestations, while behavioral abnormalities are also frequently reported (Grzonka et al., 2025). Of note, presentation may be less typical in immunocompromised patients, complicating early recognition (Tan et al., 2012). Brain imaging following HSE often reveals temporal lobe necrosis (Ak et al., 2026). Additionally, cerebrospinal fluid (CSF) typically reveals a lymphocytic pleocytosis, elevated protein concentration, and the presence of red blood cells, while detection of HSV DNA by PCR remains central to the diagnosis (Bradshaw and Venkatesan, 2016).

The prefrontal cortex (PFC), in humans, is responsible for higher-order cognitive functions such as planning, decision-making, attention, working memory, behavioral control, and problem-solving (Haber and Robbins, 2022). It is highly interconnected with several brain regions, including the hippocampus and amygdala, which contribute to memory formation, emotional processing, and the integration of contextual information (Eichenbaum, 2017). For this review, we will focus on the effects of HSV-1 on the PFC, namely the medial prefrontal cortex (mPFC) and the orbitofrontal cortex (OFC). The mPFC specializes in self-related processing and emotional or social regulation (Euston et al., 2012). It evaluates self-referential thoughts, feelings, and behaviors, and supports social cognition by correctly interpreting other people’s behavior (Wagner et al., 2012). It plays a critical part in integrating thoughts and emotions into goal-directed behavior, self-reflection, and decision-making (Hiser and Koenigs, 2018). The OFC is responsible for evaluating the consequences and value of choices to guide flexible behavior, with its primary roles including evaluation of reward versus punishment, and assigning value to everchanging context (Robin and Bastin, 2025). Lastly, the OFC is essential for impulse control, behavioral flexibility, and inhibiting inappropriate responses based on anticipated outcomes (Rudebeck and Rich, 2018). Together these regions of the PFC ensure behavioral flexibility required for daily functioning.

Although HSE is classically associated with the medial temporal lobes and limbic system, increasing clinical and neuroimaging evidence demonstrates that HSV-1-associated injury extends into the PFC, including the mPFC and OFC (Matthews et al., 2022; Rayan et al., 2022). This regional involvement may be particularly important for understanding the persistent executive, motivational, and behavioral abnormalities observed during HSE, and following supposed clinical recovery (McGrath et al., 1997; Harris et al., 2020). However, the mechanisms by which HSV-1 reaches specific prefrontal regions, the cellular processes through which infection disrupts these regions, and the relationship between regional injury and subsequent neurocognitive dysfunction remain incompletely integrated across the literature.

Accordingly, this review examines HSV-1 neuropathogenesis through a prefrontal cortical framework. We first evaluate evidence for HSV-1 involvement of specific PFC subregions and proposed mechanisms underlying their vulnerability. We then examine the cellular mechanisms of HSV-1-associated injury, including microglial and inflammasome activation, astrocytic dysfunction, neuronal injury, disruption of the neurovascular unit, and viral immune-evasion mechanisms. Lastly, we integrate these findings with the functional organization of the PFC to examine how injury to specific prefrontal regions and their associated networks may contribute to the cognitive, motivational, and behavioral sequelae of HSE.

## 2 HSV-1 tropism within the prefrontal cortex

It is well established that HSV-1 affects the medial temporal lobes and insular cortex; however, accumulating neuroimaging and clinicopathological evidence demonstrates that infection frequently extends into prefrontal cortical regions (Bello-Morales et al., 2020; Meshref et al., 2024; Ak et al., 2026). Although most available evidence consists of case reports and small imaging series rather than systematic anatomical studies, these reports consistently identify vulnerability of the mPFC and OFC to infection. Together, they suggest that HSV-1 exhibits selective vulnerability within frontal-limbic networks rather than producing diffuse frontal injury, as previously suggested.

### 2.1 Medial prefrontal cortex

Evidence for mPFC involvement is limited but demonstrates extension of HSV pathology beyond the cingulate cortex. Buerger et al. (2015) described a patient with initially isolated medial frontal cortical abnormalities and negative HSV PCR whose subsequent imaging evolved into the classic radiographic pattern of HSV encephalitis before the CSF PCR became positive. This temporal evolution suggests that medial prefrontal involvement may precede more typical temporal-lobe abnormalities in selected patients. Evidence for persistent mPFC dysfunction following HSV encephalitis was provided by Feng et al. (2026) which demonstrated reduced gray matter volume and altered hippocampal-prefrontal connectivity months after infection. This study also determined that cognitive scores were lower in patients with HSE and positively correlated with reduced gray matter volume. Although these findings do not establish direct viral infection of the mPFC, they indicate that HSV encephalitis results in long-term disruption of medial prefrontal functional networks that correlate to cognitive performance. Taken together, available evidence suggests that the mPFC participates in both acute HSV encephalitis and chronic post-encephalitic network dysfunction, although direct evidence of preferential viral tropism remains limited.

### 2.2 Orbitofrontal cortex

Compared with the mPFC, evidence for OFC involvement primarily derives from neuroimaging studies demonstrating progression of the disease within frontal-limbic networks. Ono et al. (2009) reported serial MRI findings showing spread of HSV infection from the medial temporal lobe into the OFC, basal ganglia, and thalamus during disease progression. These observations suggest that orbitofrontal abnormalities may emerge as infection extends beyond its initial temporal focus.

Similarly, James et al. (2018) demonstrated bilateral orbitofrontal, insular, and medial temporal abnormalities in PCR-confirmed HSE. Because orbitofrontal involvement occurred alongside the classic limbic distribution of HSV encephalitis, these findings support involvement of the OFC as part of broader frontal-limbic disease rather than isolated frontal infection. Additional contemporary case reports have reproduced this pattern, demonstrating orbitofrontal abnormalities together with medial temporal and insular lesions, further supporting the reproducibility of orbitofrontal involvement during HSV encephalitis (Patoulias et al., 2017). Overall, current evidence suggests that orbitofrontal abnormalities are a recurrent component of HSV-associated frontal-limbic disease, although available studies do not establish selective viral tropism for the OFC.

### 2.3 Proposed mechanisms underlying HSV-1 prefrontal cortical tropism

Although accumulating clinical evidence demonstrates recurrent involvement of the mPFC and OFC during HSV-1 encephalitis, the mechanisms responsible for this regional distribution remain incompletely understood. To date, no primary study has directly demonstrated sequential spread of naturally acquired HSV-1 from peripheral inoculation sites to specific prefrontal cortical subregions. Instead, current understanding is derived from complementary experimental systems examining peripheral neural entry, axonal transport, transsynaptic dissemination, and regional susceptibility. Collectively, these studies support a biologically plausible model of prefrontal involvement while highlighting an important gap in the literature.

Experimental studies in mice indicate that HSV-1 gains access to the CNS primarily through olfactory and trigeminal sensory pathways (Mori et al., 2005; Shivkumar et al., 2013; Dong et al., 2020). Following intranasal inoculation, HSV-1 preferentially infects olfactory sensory neurons exploiting dynein- and kinesin-dependent microtubule transport to spread to the trigeminal ganglion, establishing the olfactory neuroepithelium as an important portal of neural entry (Dong et al., 2020; Kim et al., 2024). Mori et al. (2005) further demonstrated that HSV-1 utilizes the vomeronasal chemosensory stem, spreading from the vomeronasal organ to the accessory olfactory bulb, medial amygdala, and hypothalamus through anatomically connected limb pathways. More recently, Niemeyer et al. (2024) confirmed that HSV-1 enters the CNS through both olfactory- and trigeminal-associated routes in a murine model, although cortical involvement was absent in their nonencephalitic model, indicating CNS entry alone may be insufficient to explain subsequent prefrontal infection.

Once HSV-1 has entered peripheral neurons, the virus exploits host microtubule transport machinery to reach the neuronal soma. Using cultured neurons derived from dorsal root ganglia of embryonic chickens and structural biochemical approaches, Kim et al. (2024) demonstrated that the viral tegument protein pUL37 regulates kinesin-1 activity, promoting dynein-dependent retrograde transport of incoming viral particles toward the neuronal cell body. Following productive infection, progeny virions undergo kinesin-dependent anterograde transport within cortical axons, as demonstrated by Dong et al. (2020) using fluorescent HSV-1 H129 particles in cultured murine cortical neurons. Together, these studies establish the molecular mechanisms by which HSV-1 can travel efficiently through anatomically connected neuronal pathways following peripheral infection, towards the nucleus of CNS neurons for viral production.

Experimental studies in mouse cortical neurons further suggest that HSV-1 is capable of transsynaptic dissemination. Using the anterogradely spreading HSV-1 strain H129, Qin et al. (2026) demonstrated that viral spread depends on host synaptic transmission machinery, providing direct evidence that alphaherpesviruses can propagate between synaptically connected neurons. Although H129 is a laboratory-derived tracing strain and therefore does not fully recapitulate the biology of naturally circulating HSV-1, these findings provide a plausible mechanism by which infection could extend beyond initial limbic structures into higher-order cortical networks.

Based on these findings, we speculate that prefrontal cortical involvement occurs through progressive dissemination along established frontal-limbic circuits rather than direct infection of isolated cortical regions. Following peripheral infection, HSV-1 likely enters the CNS through olfactory and trigeminal pathways before reaching interconnected limbic structures, including the piriform cortex, amygdala, entorhinal cortex, hippocampus, and insula. These regions maintain dense reciprocal projections with the mPFC and OFC through well-characterized limbic-prefrontal networks. Consequently, neuron-to-neuron spread along these existing anatomical connections provides a biologically plausible explanation for the reproducible involvement of medial and orbital prefrontal regions observed in human HSV encephalitis. This hypothesis is further supported by the previously discussed clinical neuroimaging, demonstrating that prefrontal abnormalities almost invariably occur alongside temporal, insular, or other limbic lesions rather than in isolation, consistent with progressive spread through interconnected cortical networks.

In addition to anatomical connectivity, intrinsic regional susceptibility may influence the final distribution of infection. Using stereotactic inoculation of pseudorabies virus, an alphaherpesvirus closely related to HSV-1, in mice, Korff et al. (2025) demonstrated preferential viral accumulation within medial temporal, piriform, and prefrontal cortices. Although these experiments were performed using pseudorabies virus rather than naturally acquired HSV-1 infection, they provide the strongest experimental evidence that intrinsic biological properties of prefrontal cortex may contribute to its selective vulnerability once the virus reaches the forebrain.

Taken together, available evidence supports a multistep model of HSV-1 prefrontal cortical involvement, whereby peripheral infection is followed by neural entry through olfactory or trigeminal pathways, microtubule-dependent axonal transport, transsynaptic dissemination through interconnected limbic circuits, and preferential establishment of infection within biologically susceptible cortical regions. However, this sequence remains a proposed mechanistic model rather than a directly demonstrated pathway, as no study has yet visualized continuous spread of naturally acquired HSV-1 from peripheral inoculation sites to individual prefrontal cortical subregions. Future studies combining viral tracing, high-resolution neuroimaging, and spatial transcriptomics will be required to determine why the mPFC and OFC exhibit reproducible vulnerability during HSV-1 encephalitis.

## 3 Cellular mechanisms of HSV-1 viral injury

Before discussing region-specific injury within the mPFC and OFC, we will summarize the biological mechanisms by which HSV-1 injures CNS tissue. Although most studies are not PFC-specific, these conserved pathways likely operate within infected prefrontal regions and collectively contribute to executive dysfunction, impaired working memory, emotional dysregulation, and behavioral abnormalities observed following HSV encephalitis.

### 3.1 Innate immune activation

#### 3.1.1 Microglia

Microglia are the resident immune cells of the CNS and serve as the first line of defense against neurotropic viral infection (O’Brien et al., 2022). Beyond their immune functions, microglia are essential for the development and maintenance of PFC circuits through regulation of synaptic pruning, axonal remodeling, myelination, and synaptic homeostasis (Ginhoux and Guilliams, 2016; Marinelli et al., 2019; Blagburn-Blanco et al., 2022; Choi and Chung, 2026). Following CNS infection, microglia rapidly detect viral nucleic acids and tissue injury through pattern-recognition receptors, initiating antiviral responses that limit viral dissemination while coordinating communication with astrocytes, neurons, and infiltrating immune cells (Hanisch and Kettenmann, 2007; Liu et al., 2016; Zhao et al., 2022). Although these responses are critical for early host defense, persistent activation can disrupt synaptic homeostasis, amplify neuroinflammation, and contribute to long-term neuronal dysfunction (Salter and Stevens, 2017; Huo et al., 2024; Angelovich et al., 2025).

HSV-1 provides one of the best-characterized examples of the dual role of microglia during viral encephalitis. Following entry into the CNS, HSV-1-derived double-stranded DNA is recognized by the cytosolic DNA sensor cyclic GMP-AMP synthase (cGAS), resulting in activation of the stimulator of interferon genes (STING) pathway in resident microglia (Reinert et al., 2016). cGAS synthesizes cyclic GMP-AMP (cGAMP), which activates STING and promotes interferon regulatory factor 3 (IRF3)-dependent transcription of type 1 interferons (IFN) and IFN-stimulated genes. Reinert et al. (2016) demonstrated that microglia are the principal early source of type 1 IFNs following HSV-1 brain infection and that mice lacking expression of either cGAS or STING exhibit markedly increased viral replication, susceptibility to HSE, and mortality. These findings are further supported by Katzilieris-Petras et al. (2022) who utilized mouse models to show that depletion of microglia prior to HSV-1 CNS invasion in addition to reduced IFN production also impaired early cytokine and chemokine production, promoting viral dissemination and worsened encephalitic disease. Together, these studies indicate that rapid microglial activation is required to restrict HSV-1 during the earliest stages of CNS infection.

Microglial activation, however, is not static. Rather than existing as discrete “resting” and “activated” states, microglia undergo dynamic transcriptional remodeling throughout infection. Building upon their earlier work on cGAS-STING deficient mice models, Reinert et al. (2021) showed the consequences of prolonged cGAS-STING activation was dependent on viral burden. This study elucidated that low-to-moderate viral loads favored protective type I IFN production, whereas high viral burdens trigged cGAS-STING-dependent apoptosis of microglia and other immune cells. Consistent with this concept, temporally resolved murine single-cell transcriptomic analysis by Ding et al. (2025) demonstrated that microglia are among the earliest immune populations to respond following HSV-1 brain infection, exhibiting antiviral and inflammatory transcriptional programs before substantial recruitment of circulating monocytes and T cells. As the disease advances, microglia undergo serial changes in subpopulation composition upon infection from initial homeostasis to an early IFN-mediated pro-inflammatory state in the presence of virus and eventually transitioning to proliferative and antigen-presenting states at later time points. This indicates that microglial activation represents a dynamic continuum rather than a single activation state. Collectively, these findings suggest that prolonged or overwhelming HSV-1 infection may contribute toward inflammatory phenotypes that contribute to CNS injury.

HSV-1 also actively suppresses microglial antiviral defenses. Studies have shown that HSV-1 disrupts the Golgi structural protein GM130, resulting in Golgi fragmentation, reduced TLR3 expression (which recognizes dsRNA of viral origin), diminished production of IFN-β, TNF-α, and IL-6, and subsequent increased viral replication (Botos et al., 2009; Liu J. et al., 2024). The Liu et al. study showed that restoration of GM130 partially rescued antiviral cytokine production and limited viral replication, suggesting that HSV-1 selectively suppresses microglial innate immunity rather than globally inhibiting inflammatory responses. Similarly, Idorn et al. (2026) used transgenic mice with the IRF3 R278Q mutation, ortholog to HSE-associated IRF3 R285Q, and iPSC-derived CNS cells from a pediatric patient carrying the variant to demonstrate that impaired IRF3-dependent IFN response produced a paradoxically more severe inflammatory phenotype. Their models showed that defective microglial type I IFN responses resulted in increased cerebral viral burden followed by NF-κB-enriched inflammatory immune populations and more severe neurological disease. Together, these findings suggest that inadequate early antiviral responses permit greater viral replication, ultimately driving secondary inflammatory injury.

Beyond immune defense, microglia continuously regulate synaptic remodeling, neuronal connectivity, and phagocytic clearance of dysfunctional synaptic elements. Consequently, prolonged antiviral activation has the potential to compromise these homeostatic functions. Wang et al. (2020) demonstrated that HSV-1 infection induced marked microglial activation characterized by amoeboid morphology, increased CD68-associated phagocytic activity, and NLRP3 inflammasome activation. In an Alzheimer’s disease mouse model, these changes were accompanied by increased amyloid-β deposition and worsening cognitive impairment, whereas pharmacologic inhibition of NLRP3 improved both pathology and behavioral performance (Wang et al., 2024). Although this model represents chronic HSV-1 exposure rather than acute HSE and does not specifically examine prefrontal cortical circuits, it suggests that sustained antiviral microglial activation may divert microglia from their normal homeostatic functions, thereby contributing to persistent neuronal dysfunction.

Overall, current evidence supports a model in which microglial activation evolves from an initially protective antiviral response to a potential driver of secondary neuronal injury when viral replication persists. In addition to restricting viral replication, activated microglia influence neighboring neurons and astrocytes through IFNs, cytokines, and chemokines, thereby shaping the broader cellular response to HSV-1 infection. Because microglia regulate synaptic homeostasis and coordinate interactions with astrocytes and other CNS cells, dysregulated microglial activation likely represents one of the earliest mechanisms linking HSV-1 infection to disruption of cortical networks. The downstream inflammatory pathways initiated by activated microglia, including inflammasome signaling, are discussed in the following sections.

#### 3.1.2 NLRP3 inflammasome activation

Inflammasomes are cytosolic multiprotein signaling complexes that amplify innate immune responses following recognition of microbial infection or cellular stress (Zheng et al., 2020). Activation of inflammasome sensors leads to assembly of the adaptor protein apoptosis-associated speck-like protein containing a CARD (ASC), activation of caspase-1, and subsequent maturation of the pro-inflammatory cytokines IL-1β and IL-18. Caspase-1 also cleaves gasdermin D, a cell membrane protein that can trigger pyroptosis. This is a highly inflammatory form of programmed cell death. Together, these responses help eliminate infected cells and recruit additional immune cells to sites of infection. During viral encephalitis, inflammasome activation serves a dual role. Early activation contributes to antiviral defense by limiting viral replication, whereas sustained or excessive activation promotes chronic neuroinflammation, pyroptotic cell death, and secondary neuronal injury (Yang et al., 2026). Thus, inflammasomes represent a critical transition point between protective innate immunity and immunopathology.

HSV-1 activates multiple inflammasome pathways while simultaneously encoding mechanisms to suppress their antiviral functions. This balance between inflammasome activation and viral immune evasion appears to influence both viral control and the extent of secondary inflammatory injury. As discussed, activation of the cGAS–STING pathway not only induces type I IFN responses but also promotes inflammasome signaling. Using human macrophage cell lines, primary murine immune cells, and complementary mouse models of HSV-1 infection, Wang et al. (2020) demonstrated that STING directly promotes NLRP3 inflammasome activation by recruiting NLRP3 to the endoplasmic reticulum, facilitating its deubiquitination, and enhancing caspase-1 activation and IL-1β production. These findings establish STING as a molecular bridge linking intracellular HSV-1 DNA sensing to downstream inflammasome activation and demonstrate that antiviral IFN signaling and inflammatory cytokine production function as coordinated rather than independent immune pathways.

Further, using HSV-1-infected murine BV2 microglial cells, Hu et al. (2022) demonstrated that HSV-1 activates the NLRP3 inflammasome, resulting in caspase-1 activation, IL-1β maturation, gasdermin D cleavage, and pyroptotic cell death. Pharmacologic inhibition of NLRP3 significantly attenuated pyroptosis, establishing a direct mechanistic link between HSV-1 infection and inflammasome-mediated microglial injury. Although demonstrated in an *in vitro* murine model, these findings provide a plausible mechanism through which sustained inflammasome activation could amplify neuroinflammation while impairing microglial functions necessary for antiviral surveillance and synaptic homeostasis. While not microglia, another study showed the same HSV-1-mediated inflammasome activation and pro-inflammatory cytokine production in human monocyte derived macrophages (Karaba et al., 2020). Complementing these cellular findings, Hayes et al. (2021) using NLRP3- and ASC-deficient mouse models of HSE, demonstrated that loss of inflammasome signaling significantly reduced CNS inflammation, immunopathology, and mortality. Together, these studies indicate that although NLRP3 activation contributes to antiviral immunity during acute HSV-1 infection, and persistent inflammasome signaling can become immunopathologic and contribute to secondary neuronal injury beyond direct viral cytotoxicity.

HSV-1 simultaneously limits inflammasome-mediated antiviral defense through viral immune evasion. Using human macrophages, recombinant HSV-1 VP22 deletion mutants, and complementary mouse models of HSV-1 infection, Maruzuru et al. (2018, 2021) demonstrated that the HSV-1 tegument protein VP22 directly binds the cytosolic DNA sensor absent in melanoma 2 (AIM2), preventing AIM2 oligomerization and subsequent inflammasome assembly. These findings establish VP22-mediated AIM2 inhibition as a functional mechanism of HSV-1 immune evasion that promotes viral persistence during CNS infection. Unlike NLRP3-mediated inflammation, VP22-mediated AIM2 inhibition does not directly induce tissue injury. Rather, suppression of AIM2 weakens early intracellular antiviral defense, allowing greater viral replication before broader inflammatory pathways become activated. Although these experiments were performed primarily in cultured immune cells and mouse models, this mechanism provides a biologically plausible explanation for how HSV-1 establishes infection within susceptible brain regions before widespread neuroinflammation develops.

Collectively, these studies suggest that inflammasome signaling represents a balance between antiviral protection and immunopathology. Early activation of NLRP3 contributes to viral containment, whereas persistent activation promotes cytokine release, pyroptosis, and secondary neuronal injury. Concurrent viral suppression of AIM2 further shifts this balance by permitting increased viral replication before inflammatory responses become fully established. Together, these mechanisms provide a plausible framework through which HSV-1-associated neuroinflammation may contribute to dysfunction of prefrontal cortical circuits.

### 3.2 Glial dysfunction

#### 3.2.1 Astrocytes

Astrocytes are the most abundant glial cells within the CNS and perform essential functions in maintaining neuronal homeostasis (Wei and Morrison, 2026). In addition to regulating neurotransmitter recycling, such as controlling excitatory glutamate levels to help protect neurons, metabolic support, ion homeostasis, and blood–brain barrier (BBB) integrity, astrocytes actively participate in innate immune responses following viral infection. Through expression of pattern-recognition receptors and IFN signaling pathways, astrocytes detect viral nucleic acids, produce antiviral cytokines, and coordinate communication with neighboring neurons, microglia, endothelial cells, and infiltrating immune cells (Jorgačevski and Potokar, 2023). Consequently, astrocytes serve not only as structural support cells but also as critical regulators of antiviral defense and neuroinflammation. Disruption of astrocytic function during viral encephalitis may therefore compromise neuronal homeostasis while amplifying secondary inflammatory injury.

Astrocytes constitute an essential component of the CNS antiviral response to HSV-1 infection. Using astrocyte-specific IFNAR1 knockout mice in a murine model of HSE, Acuña-Hinrichsen et al. (2021) demonstrated that astrocytes mount a robust type I IFN response following HSV-1 infection. Loss of astrocytic IFNAR1 signaling resulted in markedly increased cerebral viral replication, greater encephalitis severity, and increased mortality, demonstrating that astrocytic IFN signaling actively restricts viral dissemination within the CNS. These findings establish astrocytes as active participants in antiviral immunity rather than passive structural support cells. Complementing these animal studies, Liu Y. et al. (2024) using primary human astrocytes, demonstrated that activation of cytosolic viral DNA-sensing pathways induced IRF1-dependent IFN signaling, increased expression of antiviral IFN-stimulated genes, and substantially reduced HSV-1 replication. In this study, clustered regularly interspaced short palindromic repeats (CRISPR)-mediated disruption of IRF1 significantly impaired antiviral protection, identifying IRF1 as a central regulator of astrocytic antiviral immunity. Together, these studies indicate that astrocytes possess robust intrinsic antiviral programs that actively limit HSV-1 replication in both experimental animal models and human cells.

Beyond disrupting antiviral signaling, HSV-1 directly compromises astrocyte survival. Using human stem cell-derived brain organotypic slice cultures, Rashidi et al. (2024) demonstrated that astrocytes are productively infected by HSV-1 and undergo regulated necroptotic cell death alongside infected neurons. Complementing these findings, Jeffries et al. (2022), using cultured murine astrocytes and experimental HSV-1 infection models, demonstrated that HSV-1 activates ZBP1- and RIPK3-dependent signaling, triggering both necroptotic and apoptotic astrocyte death. This loss of astrocytes leads to impaired antiviral responses and disrupted maintenance of neuronal homeostasis. Collectively, these studies indicate that HSV-1 not only infects astrocytes but actively eliminates them through regulated cell-death pathways, thereby compromising metabolic support, antiviral immunity, and homeostatic tasks essential for normal CNS function.

Evidence from human primary astrocytes, human organotypic brain models, and complementary murine studies demonstrates that astrocytes play an essential role in limiting HSV-1 dissemination while maintaining neuronal homeostasis during CNS infection. Failure of astrocytic IFN signaling permits increased viral replication, whereas regulated astrocyte death further compromises metabolic support, inflammatory regulation, and communication with neighboring neurons and microglia. Although these astrocytic mechanisms have been characterized primarily in human astrocytes and experimental animal models rather than within individual prefrontal cortical subregions, clinical case reports and neuroimaging studies demonstrating HSV-1 involvement of the mPFC and OFC support the biological plausibility that similar astrocytic responses occur within infected prefrontal tissue (Kapur et al., 1994; Taylor et al., 2007; James et al., 2018; Feng et al., 2026; Ngan et al., 2026). Consequently, astrocyte dysfunction provides a biologically plausible mechanism through which HSV-1 infection may contribute to disruption of prefrontal cortical networks underlying executive function, motivation, working memory, and behavior.

#### 3.2.2 Blood–brain barrier and neurovascular unit

The BBB is formed by a specialized neurovascular unit (NVU) composed of brain microvascular endothelial cells, astrocyte end feet, pericytes, neurons, and microglia (Dotiwala et al., 2026). Together, these cells regulate vascular permeability, maintain CNS immune privilege, and coordinate communication between the circulation and the brain. Rather than functioning as an isolated endothelial barrier, the NVU operates as an integrated multicellular network in which glial cells actively regulate endothelial integrity through cytokine signaling, metabolic support, and immune surveillance. Consequently, disruption of glial homeostasis may impair BBB function, facilitate peripheral immune-cell infiltration, and amplify neuroinflammation during viral encephalitis.

Maintenance of BBB integrity during HSV-1 infection depends heavily on coordinated antiviral signaling between astrocytes and microglia (discussed earlier). Impaired astrocytic IFN signaling and defective microglial antiviral responses increase viral dissemination and inflammatory cytokine production, creating conditions that compromise neurovascular homeostasis. Direct evidence for this mechanism was provided by Zhang et al. (2025) using a three-dimensional human neurovascular unit model consisting of brain microvascular endothelial cells, astrocytes, microglia, and neurons. They demonstrated that HSV-1 infection caused severe suppression of glial autophagic flux, increased inflammatory cytokine production, endothelial dysfunction, BBB breakdown, and peripheral immune-cell infiltration despite minimal direct infection of endothelial cells. Interestingly, restoration of autophagy reduced viral replication and improved neurovascular integrity, suggesting that BBB disruption results primarily from glial dysfunction rather than direct endothelial infection.

Collectively, these findings indicate that HSV-1-associated BBB dysfunction is an indirect consequence of dysregulated glialvascular interactions. Rather than directly targeting endothelial cells, HSV-1 impairs the coordinated antiviral functions of astrocytes and microglia, leading to inflammatory cytokine production, increased vascular permeability, and infiltration of peripheral immune cells. Although these mechanisms have not been examined specifically within individual prefrontal cortical subregions, disruption of the neurovascular unit provides a plausible mechanism through which sustained neuroinflammation may contribute to dysfunction of prefrontal cortical networks following HSV encephalitis.

### 3.3 Neuronal injury

Neurons are the principal functional targets of neurotropic viral infection and are essential for maintaining the synaptic transmission and network connectivity underlying higher-order cognitive processes. Within the PFC, neuronal integrity is required for executive function, working memory, decision-making, and behavioral regulation. Viral infection can impair neuronal function through multiple mechanisms, including direct cytopathic injury, disruption of synaptic architecture, altered transcriptional regulation, mitochondrial dysfunction, impaired autophagy, and activation of regulated cell-death pathways (Wongchitrat et al., 2024). Importantly, accumulating evidence suggests that neuronal dysfunction frequently precedes overt neuronal loss, indicating that alterations in neuronal physiology and connectivity may contribute to persistent dysfunction of prefrontal cortical networks that may underlie executive, cognitive, and behavioral deficits following viral encephalitis.

Rather than simply inducing neuronal death, HSV-1 profoundly alters neuronal function early during infection. Using primary murine cortical neurons infected with HSV-1, Acuña-Hinrichsen et al. (2021) demonstrated marked dendritic spine loss, degradation of the postsynaptic scaffolding protein PSD-95, disruption of postsynaptic density organization, abnormal calcium signaling, and impaired glutamatergic neurotransmission. This study also determined an HSV-1-mediated upregulation and accumulation of the synaptic hub protein Arc within the soma. Interestingly, despite elevated Arc levels, its ability to interact with postsynaptic scaffolding proteins underwent a progressive decline and ultimately ceased. The authors hypothesized that the viral kinase US3 is responsible for Arc accumulation, however further research is required. Regardless, the structural collapse interfered with glutamate receptor membrane anchoring, rendering the infected neurons completely unresponsive to glutamate stimulation. These findings indicate that HSV-1 compromises synaptic architecture and neuronal connectivity independently of overt cytotoxicity, suggesting that functional impairment is an early feature of infection in part due to synaptic dysfunction and aberrant behavior in neuronal networks.

In addition to structural changes, HSV-1 extensively reprograms neuronal gene expression. González-Almela et al. (2025) used primary human neuronal cultures to demonstrate that HSV-1 extensively reprograms neuronal transcription through redistribution of RNA polymerase II and cohesin, resulting in higher-order chromatin reorganization and suppression of host neuronal gene expression without widespread destruction of chromatin architecture. This transcriptional remodeling altered expression of neurotransmitter receptors, ion channels, synaptic proteins, and plasticity-associated genes, suggesting that HSV-1 reshapes the neuronal transcriptional landscape. Together, these studies suggest that both synaptic disassembly and transcriptional reprogramming contribute to neuronal dysfunction before irreversible cell loss.

HSV-1 further impairs neuronal survival by disrupting mitochondrial homeostasis and autophagy. Using cultured neurons from human foreskin fibroblasts, human embryonic kidney cells, and African green monkey kidney cells infected with wild-type and mutant HSV-1 strains, Manivanh et al. (2020) demonstrated that the viral neurovirulence infected cell protein 34.5 (ICP34.5) alters mitochondrial trafficking and morphology, promotes oxidative stress, and interacts with phosphoglycerate mutase family member 5 (PGAM5). Interaction with PGAM5, a mitochondrial phosphatase involved in mitochondrial homeostasis and regulated cell-death signaling, lead to oxidative stress and helped facilitate viral replication. Earlier work by Orvedahl et al. (2007) established ICP34.5 that suppresses neuronal autophagy through Beclin-1 binding, thereby overcoming an important intrinsic antiviral defense. The molecular mechanisms underlying these effects are discussed further in Section 3.4.

Although neuronal dysfunction develops early during HSV-1 infection, progressive neuronal loss also contributes to CNS injury. Using human organotypic brain slice cultures together with human HSE tissue, Rashidi et al. (2024) demonstrated that HSV-1 induces regulated necroptotic cell death of both neurons and astrocytes rather than nonspecific viral cytotoxicity. Similar patterns of cortical injury observed in human HSE tissue suggest that regulated cell-death pathways contribute substantially to neuropathology and may prolong neuroinflammation beyond the period of active viral replication.

Collectively, these findings indicate that HSV-1-associated neuronal injury evolves through a continuum of functional and structural changes. Early disruption of synaptic organization, neuronal transcription, mitochondrial homeostasis, and autophagy precedes regulated neuronal death, suggesting that neuronal dysfunction develops well before irreversible cell loss. These mechanisms provide a plausible basis for the persistent disruption of cortical networks underlying executive function, working memory, and behavioral regulation following HSE.

### 3.4 Viral immune evasion

Unlike the preceding sections, which focus primarily on host responses to infection, viral immune-evasion proteins represent pathogen-driven mechanisms that actively manipulate host biology to facilitate HSV-1 infection within the CNS. HSV-1 encodes multiple proteins that suppress innate immune sensing, interfere with antiviral signaling, reprogram host transcription, and disrupt cellular homeostatic pathways to create an intracellular environment favorable for viral replication and persistence. For a thorough review on HSV-1 immune evasion strategies, see Zhu and Zheng (2020). These HSV-1 viral factors do not act independently of the cellular mechanisms described above; rather, they shape their initiation, magnitude, and persistence by simultaneously promoting viral replication and attenuating host antiviral defenses. Thus, HSV-1 neurovirulence reflects an interaction between direct viral manipulation of host cells and the secondary microglial, inflammasome, astrocytic, neuronal, and neurovascular responses that follow.

HSV-1 neurovirulence is mediated by the coordinated actions of several viral proteins that target complementary host pathways. Among these, ICP34.5 is considered the principal determinant of HSV neurovirulence. Using primary murine cortical neurons and mouse models, Orvedahl et al. (2007) demonstrated that ICP34.5 binds Beclin-1 and suppresses neuronal autophagy. HSV-1 mutants lacking the Beclin-1-binding domain in ICP34.5 failed to inhibit autophagy and exhibited markedly reduced neurovirulence, establishing autophagy inhibition as a fundamental determinant of HSV neuropathogenesis. Building upon these findings, Manivanh et al. (2020) using primary neuronal cultures, demonstrated that ICP34.5 additionally interacts with the mitochondrial PGAM5 to alter mitochondrial trafficking, increase oxidative stress, and facilitate viral replication. These observations broaden the role of ICP34.5 beyond autophagy inhibition, suggesting that HSV simultaneously contributing to the neuronal dysfunction described in Section 3.3.

HSV-1 also promotes viral innate immune suppression. As discussed earlier, Maruzuru et al. (2018) using human macrophages, recombinant HSV-1 VP22 deletion mutants, and complementary mouse infection models, demonstrated that the tegument protein VP22 binds AIM2, preventing AIM2 oligomerization and subsequent inflammasome assembly. Deletion of VP22 restored AIM2-dependent IL-1β and IL-18 production while significantly reducing viral replication *in vivo*, demonstrating that suppression of AIM2 provides a functional advantage during CNS infection. A subsequent study further demonstrated that the DNA-binding activity of VP22 is required for efficient AIM2 antagonism, providing a molecular explanation for how VP22 suppresses intracellular DNA sensing during HSV infection (Maruzuru et al., 2021).

In addition to disrupting innate immunity, HSV-1 reprograms host transcription to favor viral gene expression. Using primary neuronal cultures infected with HSV-1, Liu S. et al. (2024) demonstrated that the viral protein infected cell protein 22 (ICP22) binds the facilitates chromatin transcription (FACT) complex, increasing RNA polymerase II occupancy on viral genes while suppressing host transcription. Mutations disrupting the ICP22–FACT interaction significantly reduced viral gene expression, replication, and neurovirulence, demonstrating that HSV actively hijacks neuronal chromatin machinery to sustain productive infection.

A 2013 study revealed that HSV neurovirulence extends beyond these well-characterized proteins. UL36, an HSV-1 ubiquitin-specific protease, was determined to be a novel neurovirulence factor required for efficient viral replication within the CNS (Wang et al., 2013). UL36 is required for efficient CNS infection through mechanisms that remain incompletely defined, however it seems that by deubiquitinating TRAF3, the IFN pathway is dysregulated, resisting an otherwise IFN-driven antiviral state.

Collectively, these viral proteins function cooperatively to establish an intracellular environment favorable for persistent infection. By suppressing autophagy, evading innate immune sensing, reprogramming host transcription, and adapting to the neuronal metabolic environment, HSV-1 sustains viral replication while promoting dysregulated immune activation, glial dysfunction, neuronal injury, and neurovascular disruption. Viral immune evasion therefore represents a unifying mechanism that links HSV-1 persistence to progressive CNS injury.

## 4 Linking regional injury to clinical dysfunction

HSV-1 employs multiple immune-evasion and neurovirulence mechanisms that facilitate viral replication while disrupting host pathways required for neuronal and glial homeostasis. Together with the microglial, inflammasome, astrocytic, neuronal, and neurovascular responses described above, these mechanisms provide a cellular framework for HSV-1-associated CNS injury. However, the functional consequences of this injury depend not only on its underlying mechanism, but also on its regional distribution. Given the functional specialization of the PFC, injury to distinct prefrontal subregions may therefore help explain the characteristic cognitive, motivational, and behavioral abnormalities observed during and following HSV-1 encephalitis (Ak et al., 2026).

### 4.1 Medial prefrontal cortex

The mPFC participates in the integration of emotional, motivational, contextual and mnemonic information that is required for adaptive decision-making and social behavior. These functions provide a framework for interpreting the behavioral and psychiatric abnormalities observed following HSV-1 encephalitis. Long-term clinical studies have documented executive dysfunction, depression, anxiety, and behavioral changes among HSE survivors, although these abnormalities have not been specifically localized to the mPFC and frequently occur in the setting of broader frontal and limbic injury (Harris et al., 2020). Thus, while the clinical phenotype is consistent with disruption of mPFC-associated function, existing evidence does not establish a direct relationship between mPFC injury and individual neuropsychiatric outcomes.

The strongest HSE-specific evidence for this relationship comes from Caparros-Lefebvre et al. (1996) who prospectively evaluated 11 patients with clinically presumed HSE using neuropsychological and psychiatric assessment, MRI volumetry, and single-photon emission computed tomography (SPECT). Long-term behavioral abnormalities, including emotional lability, irritability, anxiety, and depression, were prominent in seven patients, while left prefrontal hypoperfusion was identified in eight and was associated with psychiatric abnormalities in seven. Left prefrontal hypoperfusion also frequently occurred alongside left amygdala injury, leading the authors to implicate dysfunction of amygdalo-frontal pathways in persistent psychiatric and behavioral sequelae. Although SPECT localization did not isolate a specific mPFC subdivision, this study provides substantially stronger evidence than symptom reports alone since frontal physiological dysfunction and behavioral abnormalities were measured within the same HSE cohort.

Additional clinical radiologic observation provides additional support for disruption of medial frontal-limbic network follow HSE. Geffen et al. (2008) reported a 25-year-old HSE survivor with bilateral damage involving medial, ventral frontal, and temporal regions and their associated connections. Three years after infection, the patient demonstrated severe global amnesia together with marked impairment of social cognition, despite preservation of several other cognitive abilities. This is indicative of the way in which behavior and memory may be altered due to the interconnected medial and ventral frontal-limbic circuits.

Persistent psychiatric symptoms are also evident in larger HSE cohorts, where Harris et al. (2020) found that depression, anxiety, and subjective cognitive complaints remained elevated during follow-up even as several objective neuropsychological measures improved. While these findings are not anatomically specific to the mPFC, they reinforce the clinical importance of persistent affective dysfunction after HSE. When considered alongside the prefrontal hypoperfusion and amygdala injury observed by Caparros-Lefebvre et al. (1996) they support a network-level model in which HSV-associated disruption of medial prefrontal-limbic interactions may contribute to persistent abnormalities in emotional regulation and adaptive behavior.

### 4.2 Orbitofrontal cortex

As discussed, the OFC is partly responsible for reward evaluation and punishment and behavior inhibition and adaptation (Harris et al., 2020). MRI studies have shown that HSE causes orbitofrontal abnormalities alongside medial temporal and insular lesions (Ono et al., 2009; James et al., 2018). The OFC’s interconnectedness with limbic structures may be partly responsible for behavioral dysregulation when HSV-1 infection affects these areas. Clinical evidence has shown an association between HSV-1 encephalitis and changes in behavior, judgment, motivation, and executive function. Del Grosso Destreri et al. (2002) describe an HSE patient who developed confabulatory and dysexecutive syndrome and memory impairment. Similarly, Caparros-Lefebvre et al. (1996) followed 11 patients with HSV-1 encephalitis who reported cognitive and psychiatric abnormalities. Their findings suggested disruption of amygdalo-frontal pathways and temporal-limbic injury. In a similar fashion, Baldivia et al. (2008) reported extensive bilateral injury involving the orbitofrontal, insular, and medial temporal cortices following HSE, accompanied by perseverative behavior and confabulation in addition to severe memory dysfunction. When taken together, these findings support the role of the orbitofrontal and ventromedial frontal circuits in emotional regulation, decision-making, and behavioral inhibition, and the disruptions caused by HSV-1.

It must be cautioned that the behavioral abnormalities observed in post-infectious HSV-1 encephalitis patients cannot confidently be exclusively linked to OFC injury. This is because of the overlap in function of the temporal lobes, amygdala, hippocampus, insula, cingulate cortex, and other frontal regions. This information is best interpreted as supporting an all-encompassing frontal-limbic network rather than an exclusive OFC injury. It is essential this distinction is noted because the main evidence for OFC involvement is found in case studies and neuroimaging rather than studies directly linking OFC injury to clinical dysfunction. Further work to elucidate PFC-specific damage that can only be explained with HSV-1 infection and subsequent HSE-mediated CNS injury is needed.

### 4.3 Integrated model

Although distinct PFC subregions demonstrated involvement in HSE, their functional consequences cannot be considered in isolation. The mPFC and OFC are extensively connected with one another and with the hippocampus, amygdala, insula, and medial temporal structures that are commonly affected during HSE (Ak et al., 2026). Consequently, regional PFC injury may contribute to distinct components of the clinical phenotype while simultaneously disrupting broader frontal-limbic networks. Within this framework, mPFC dysfunction may contribute to altered emotional regulation and adaptive behavior, while OFC injury may contribute to impaired behavioral regulation and decision-making. However, overlap among these functions and the widespread nature of HSE make one-to-one lesion-symptom relationships unlikely. Thus, the neurobehavioral manifestations of HSE are best understood as arising from HSV-1 involvement of functionally specialized PFC regions within interconnected frontal-limbic networks.

## 5 Knowledge gaps and future directions

### 5.1 Regional mechanisms of PFC vulnerability

Despite evidence that HSV-1 can involve the mPFC and OFC, the mechanisms underlying this regional distribution remain poorly defined. Experimental studies support olfactory and trigeminal neuroinvasion, axonal and transsynaptic spread, and potential differences in regional cellular permissiveness; however, no study has directly demonstrated a continuous pathway from peripheral HSV-1 infection to individual PFC subregions. Moreover, most mechanistic studies of microglial activation, inflammasome signaling, astrocytic dysfunction, neuronal injury, and neurovascular disruption use whole-brain, non-PFC, or *in vitro* models. Thus, while inferences can be made, it remains to be established whether these processes differ across individual prefrontal regions.

### 5.2 Linking regional injury to clinical phenotype

Although HSE is known to produce executive, motivational, and behavioral dysfunction in survivors, direct relationships between specific PFC lesions and these outcomes remains insufficiently characterized. Individual reports associate cingulate or medial frontal abnormalities with apathy, abulia, impaired initiation, and executive dysfunction, but large-scale lesion-symptom mapping has not established whether mPFC or OFC injury independently predicts these neurobehavioral outcomes. Furthermore, since HSV-1 commonly affects interconnected temporal, insular, and limbic structures, it remains unclear whether these deficits arise from focal PFC injury or disruption of broader frontal-limbic networks.

### 5.3 Mechanisms of persistent dysfunction

The mechanisms underlying persistent neurocognitive impairment after control of acute HSV-1 infection also remain unresolved. Long-term dysfunction may reflect irreversible neuronal loss, persistent glial or inflammatory abnormalities, synaptic dysfunction, neurovascular injury, or maladaptive network reorganization. Whether structurally preserved prefrontal tissue remains functionally impaired after acute HSE is poorly understood. Distinguishing fixed structural injury from potentially reversible cellular and network dysfunction may have important implications for recovery and treatment.

### 5.4 Future directions

Future studies should integrate regional viral localization, cellular mechanisms, network function, and clinical outcomes within the same experimental or clinical framework. Spatial transcriptomics, single-cell approaches, and region-specific viral mapping could determine why particular PFC regions are vulnerable and whether cellular responses differ across the mPFC and OFC. In parallel, longitudinal human studies combining structural and functional neuroimaging with standardized neuropsychological testing could determine whether specific patterns of PFC and frontal-limbic network injury predict persistent cognitive and behavioral outcomes. Ultimately, connecting viral localization with cellular injury, circuit dysfunction, and clinical phenotype will be necessary to identify mechanisms and therapeutic targets responsible for long-term neurological morbidity following HSE.

## 6 Conclusion

HSE is classically characterized by medial temporal and limbic involvement; however, accumulating evidence demonstrates that the PFC is also an important component of its neuropathology. Recurrent involvement of the medial prefrontal and orbitofrontal cortices suggests that HSV-1-associated frontal injury occurs within anatomically interconnected frontal-limbic networks rather than as diffuse cortical involvement. Although the mechanisms underlying this regional vulnerability remain incompletely defined, current evidence supports contributions from neural routes of viral dissemination, circuit connectivity, and regional cellular susceptibility. HSV-1 produces injury through interacting viral- and host-mediated mechanisms, including microglial and inflammasome activation, astrocytic dysfunction, neuronal and synaptic injury, neurovascular disruption, and viral manipulation of innate antiviral defenses. These processes provide a mechanistic framework through which regional infection may disrupt prefrontal networks supporting executive function, motivation, decision-making, and behavioral regulation. However, direct relationships between specific PFC subregions, cellular mechanisms, and clinical phenotypes remain insufficiently established. A more integrated understanding of HSV-1 neuropathogenesis will therefore require moving beyond the independent study of viral localization, cellular injury, and neurocognitive outcomes. Defining how these processes converge within specific prefrontal circuits may clarify the mechanisms underlying persistent cognitive and behavioral morbidity following HSE and identify targets for preserving neurological function beyond control of acute viral replication.
